# Subacute Unilateral Bulbar Palsy

**Published:** 1894-03

**Authors:** 


### SUBACUTE UNILATERAL BULBAR PALSY.

At the meeting of the New York Neurological Society, held December 5, 1893 (*Medical Week*), Dr. Alfred Wiener reported a case of subacute unilated bulbar palsy, with autopsy, from which he concludes: 1. That the region of the hypoglossal nucleus gives origin to nerve fibers which supply the tongue, palate, pharynx and larynx on one side of the body. 2. The column of nerve fibers known as the respiratory bundle consists of fibers from the glosso-pharyngeal, vagus and vago-accessory nerve, and the lower and anterior portion of this column is probably formed of the fibers of the vagus and vago-accessory. 3. The glosso-pharyngeal nerve seems to control the reflexes of nausea and gagging in the soft palate and pharynx, and also to send a few filaments to the pharyngeal muscles. These latter filaments take their origin in the hypoglossal nucleus and ascend in the respiratory column to the nucleus proper and then make their exit with the glosso-pharyngeal nerve. 4. The soft palate muscles are not innervated by fibers from the seventh nerve.

The patient was a boy of seventeen, whose family history was negative. A few months prior to November, 1892, he had tubercular enlargement of the glands of the neck, which were successfully removed by surgical operation, but contrary to expectation nothing of a tubercular nature was found in the degeneration of the various masses of nerve cells in the medulla oblongata, which caused the symptoms and formed a basis for the conclusion above noted.

The first symptoms noted in November, 1892, were deviation of the tongue to the right side and some difficulty in swallowing, hoarseness and difficulty in coughing soon appeared, and ten days later there was complete paralysis of the right side of the tongue, soft palate, pharnyx and right recurrent laryngeal nerve. Excepting for some increasing weakness there was no change till March 29th, when an attack of respiratory failure occurred from which he recovered partially, some respiratory difficulty remaining, excessive salivation and ability to speak in a weak whisper only. Labial movements remained normal and so did the left side of the palate and pharynx. On the 20th of April an attack of respiratory failure proved fatal.

The autopsy showed disease of the nuclei of the twelfth, marked on the right, and slight on the left. The neuclei of the ninth, tenth and eleventh nerves were slightly affected, a little more on the right than on the left side. The respiratory bundle appeared completely degenerated on the right side, while on the left in the region of the hypoglossal nucleus its lower and anterior portions were diseased. The rest of the intra-medullary fibers of the ninth, vagus, and vago-accessory and hypoglossal nerves were less prominent on the right side than on the left. Otherwise, everything appeared to be perfectly natural.—*N. Amer. Prac.*

